# Exhaled breath condensate sampling is not a new method for detection of respiratory viruses

**DOI:** 10.1186/1743-422X-8-98

**Published:** 2011-03-04

**Authors:** Lieselot Houspie, Sarah De Coster, Els Keyaerts, Phouthalack Narongsack, Rikka De Roy, Ive Talboom, Maura Sisk, Piet Maes, Jannick Verbeeck, Marc Van Ranst

**Affiliations:** 1 Laboratory of Clinical Virology, Rega Institute for Medical Research, Catholic University of Leuven, Minderbroedersstraat 10, B3000 Leuven, Belgium; 2 Medical Center, Catholic University of Leuven, Naamsestraat 80, B3000 Leuven, Belgium; 3 Medical Doctor, Goudsbloemstraat 4, B3000 Leuven, Belgium

## Abstract

**Background:**

Exhaled breath condensate (EBC) sampling has been considered an inventive and novel method for the isolation of respiratory viruses.

**Methods:**

In our study, 102 volunteers experiencing upper airway infection were recruited over the winter and early spring of 2008/2009 and the first half of the winter of 2009/2010. Ninety-nine EBCs were successfully obtained and screened for 14 commonly circulating respiratory viruses. To investigate the efficiency of virus isolation from EBC, a nasal swab was taken in parallel from a subset of volunteers. The combined use of the ECoVent device with the RTube™ allowed the registration of the exhaled volume and breathing frequency during collection. In this way, the number of exhaled viral particles per liter air or per minute can theoretically be estimated.

**Results:**

Viral screening resulted in the detection of 4 different viruses in EBC and/or nasal swabs: Rhinovirus, Human Respiratory Syncytial Virus B, Influenza A and Influenza B. Rhinovirus was detected in 6 EBCs and 1 EBC was Influenza B positive. We report a viral detection rate of 7% for the EBCs, which is much lower than the detection rate of 46.8% observed using nasal swabs.

**Conclusion:**

Although very promising, EBC collection using the RTube™ is not reliable for diagnosis of respiratory infections.

## Background

Human respiratory tract infections represent the most commonly encountered infections worldwide. In the majority of cases, the etiology of these infections remains undetermined due to rapid convalescence after infection. Respiratory tract infections in healthy adults can be caused by a variety of pathogens and the detection of these agents is currently based on their isolation from nasal swabs (NS), bronchoalveolar lavages (BAL), nasopharyngeal aspirates and sputum samples. The acquisition of these specimens by semi-invasive and invasive techniques is often unpleasant for the patient. Therefore, exhaled breath condensate (EBC) analysis has recently been explored as a new and non-invasive method to monitor lung inflammation and pulmonary disease such as chronic obstructive pulmonary disease (COPD), asthma, cystic fibrosis, lung cancer etc. EBCs mainly consist of water vapour but a small fraction contains respiratory droplets derived from the airway lining fluid [1,2]. This observation has created a growing interest in the use of EBC as a new sampling method for the screening of respiratory viruses infecting the upper airways. At first, investigators suspected that turbulence of the inhaled air was responsible for the aerosolisation of the respiratory fluid. However, the effect of the turbulent airflow is limited to the upper airways since the turbulent airflow becomes laminar as it reaches the smaller bronchial airways and alveoli. Recently, the bronchiole fluid film burst model has been described [3]. This model suggests that aerosols are produced during inhalation by the bursting of fluid bubbles present in the bronchioles.

The aim of this study was to investigate whether the EBC collection method was suited for the efficient condensation of aerosolised virus particles during normal breathing and to explore the isolation of respiratory viruses in the condensate. Therefore we screened the EBC samples with virus specific PCR assays targeting 14 respiratory viruses (Human Respiratory Syncytial Virus (HRSV) A & B, Influenzavirus (Inf) A & B, Coronavirus (CoV) NL63, E229 and OC43, Adenovirus (AdV), Human Metapneumovirus (HMPV), Rhinovirus (RV) and Parainfluenza virus (PIV) 1-4).

## Methods

### Sample collection

In this study, 102 EBCs were collected from otherwise healthy volunteers showing respiratory or flu-like symptoms (defined in Table 1), using a commercially available condenser (RTube™, Respiratory Research Inc., Charlottesville, Virginia, USA). The patient was instructed to breath orally at tidal volumes into a mouthpiece attached to a condenser for 10 minutes. No nose clips were used during collection and saliva contamination was avoided by the presence of a one-way valve and the T-shaped section of the mouthpiece.

**Table 1:** Clinical manifestations of subjects

Symptoms	Serious	Mild	none
Fever	9 (8.8)	23 (22.5)	70 (68.6)
Headache	4 (3.9)	46 (45.1)	52 (51)
Shivering	7 (6.9)	27 (26.5)	68 (66.7)
Sneezing	43 (42.2)	41 (17.6)	18 (40.2)
Sore throat	38 (37.3)	34 (33.3)	30 (29.4)
Muscle ache	6 (5.9)	32 (31.4)	64 (62.7)
Watering eyes	7 (6.9)	27 (26.5)	68 (66.7)
Runny/stuffy nose	47 (46.1)	37 (36.3)	18 (17.6)
Coughing without chestpain	25 (24.5)	32 (31.4)	45 (44.1)
Coughing with chestpain	11 (10.8)	9 (8.8)	82 (80.4)

Figures represent the number of patients experiencing these clinical manifestations. Figures between brackets are the percentages calculated to total of 102 subjects.

In a first part of the study that started during the winter and spring of 2008/2009, 70 EBC samples were collected from patients who voluntary presented themselves to our laboratory. The majority of these volunteers were students that responded to the information leaflet, distributed in the university buildings of the Catholic University of Leuven. The samples were collected with the aluminium cooler sleeve chilled at -80°C.

In the fall and first half of the winter of 2009/2010, 32 condensates were collected from patients who presented themselves to their general practitioner. Due to practical circumstances, the condensates were collected with the cooler chilled at -20°C. For 13 out of 32 collections, the RTube™ was connected by a custom made connecting-piece to the ECoVent (Jaeger, Germany). This device registers ventilatory parameters such as the exhaled volume, breathing frequency and tidal volume. Additionally, a NS was obtained in parallel with the condensate collection from each patient.

All EBCs were immediately stored at -20°C. Nasal swabs (NS) were refrigerated. After viral DNA and RNA extraction, EBC samples and nasal swabs were stored at -80°C. Three specimens were excluded from the study due to incorrect condensate collection. A short questionnaire was used to document the date of birth, the severity of respiratory complaints and to record the days of symptomatic illness from all volunteers. This study was approved by the Medical Ethics Committee of the University Hospital of Leuven and informed consents were received from all participants.

### Viral RNA and DNA extraction

Viral DNA and RNA were isolated with the QIAamp MinElute Virus kit (Qiagen, Westburg, The Netherlands) according to the instruction manual. EBC extracts were eluted in 60 μl elution buffer and NS extracts in 110 μl elution buffer.

### Virus detection and sequencing

The breath condensates were screened for 11 respiratory RNA viruses (CoV NL63, E229 and OC43, RV, HMPV, InfA&B and PIV1-4) [4-7] using a OneStep RT-PCR Kit (Qiagen, Westburg, The Netherlands) in a 50 μl reaction containing 10 μl of the extracted RNA, 0.6 μM of forward and reverse primers (Table 2), 1.5 μl One Step Enzyme Mix, 10 μl 5 × One Step RT-PCR Buffer and 400 μM of each dNTP. For adenovirus screening, a DNA PCR was carried out for which the amplification reaction mix contained 0.5 μM forward primer (AdFW) and reverse primer (AdRV), 0.4 mM dNTPs, 10 μl Buffer C and 1 U Taq polymerase in a final volume of 50 μl. The PCR primers used were located in conserved regions of the genomes of the respiratory pathogens (Table 2). The reactions were carried out in a T3000 Thermocycler 48 (Westburg, Leusden, The Netherlands) with an initial reverse transcription step for RNA viruses at 50°C for 30 min, followed by PCR activation at 95°C for 30 s, 45 cycles of amplification followed by a final extension step for 10 min at 72°C. The DNA amplification program was initiated with a denaturation step at 94°C for 3 min, followed by 45 cycles of 94°C for 30 s, 55°C for 30 s and a final extension step at 72°C for 1 min. The amplicons were subjected to a 6% polyacrylamide gel and visualised under UV light by staining with ethidium bromide. PCR products were purified using the Invitek MSB Spin PCRapace Kit and cycle sequenced in forward and reverse direction using the ABI PRISM BigDye Termination Cycle Sequencing Ready Reaction kit (Applied Biosystems, Foster City, CA, USA). Sequence analysis was performed with the ABI3130 Genetic Analyser (Applied Biosystems, Foster City, CA, USA). Consensus sequences were obtained using the SeqMan II software (DNASTAR, Madison, Wis.). For samples from patient 71 and 80, rhinovirus sequences were poor, but sufficient to verify the virus by BLAST search. In addition, the VP4/VP2 partial region was amplified to confirm rhinovirus infection (data not shown). The nucleotide sequences have been deposited to GenBank under accession numbers [HM74039 to HM747056].

**Table 2:** Primers for viral screening

Primer or probe	Gene	Position	Sequence (5'→ 3')	Reference
**HRSV**				

*RT-PCR primers*				
HRSV-AF	F	669-695	ctgtgatagarttccaacaaaagaaca	[8,9]
HRSV-AF	F	718-745	agttacacctgcattaacactaaattcc	[8,9]
HRSV-BN	N	435-458	ggctccagaatataggcatgattc	[8,9]
HRSV-BN	N	480-508	tggttattacaagaagagcagctatacacagt	[8,9]
*MGB probes*				
HRSV-A TP	F	697-715	cagactactagagattacc	[8,9]
HRSV-B TP	N	461-477	tatcatcccacagtctg	[8,9]

**INFLUENZA A**				

AM_FW155	M	151-174	catggaatggctaaagacaagacc	[7]
AM_RV397	M	374-397	aagtgcaccagcagaataactgag	[7]

**INFLUENZA B**				

BHA_FW188	HA	188-209	agaccagagggaaactatgccc	[7]
BHA_RV347	HA	324-347	ctgtcgtgcattataggaaagcac	[7]

**PARAINFLUENZA**				

PIV1FW	HN	748-768	ccttaaattcagatatgtat	[4]
PIV1RV	HN	1206-1225	gataaataattattgatacg	[4]
PIV2 FW	HN	803-822	aacaatctgctgcagcattt	[4]
PIV2 RV	HN	1291-1310	argtcagacaatgggcaaat	[4]
PIV3 FW	HN	762-781	ctgtaaactcagacttggta	[4]
PIV3 RV	HN	1220-1239	tttaagcccttgtcaacaac	[4]
PIV4 FW	P	531-552	gaaagaggcttgggttacaca	[21]
PIV4 RV	P	1147-1168	gctcttatcacagtctccaaa	[21]

**HMPV**				

HMPV_N3F	N	357-379	gagaagagctgggtagaa	[6]
HMPV_N3R	N	716-733	caaacaaactttctgct	[6]

**CORONA**				

PanCoV FW	RdRp	13717-13739*	acwcarhtvaayytnaartaygc	[5]
PanCoV RV	RdRp	13948-13967*	tcrcayttdggrtartccca	[5]

**ADENO**				

AdFW	Hexon	21-46	gccscartggkcwtacatgcacatc	[22]
AdRV	Hexon	301-322	cagcacsccicgratgtcaaa	[22]

**RV**				

RV_5UTR_FW	5'UTR	186-203	caagcacttctgtttccc	[23]
RV_5UTR_RV	5'UTR	566-584	cacggacacccaaagtagt	[23]

*Nucleotide positions for reference strain CoV NL63 (DQ445912)

HRSV was detected using a RT-PCR assay as previously described [8,9]. In brief, a multiplex mix was prepared in a final volume of 25 μl using 5 μl extracted RNA, 12.5 μl of Eurogentec One-Step Reverse Transcriptase qPCR Master Mix containing ROX as a passive reference, 0.125 μl Euroscript + RT & RNase inhibitor (Eurogentec, Seraing, Belgium) 200 nM of HRSV-A and -B specific forward and reverse primers and 100 nM of HRSV-A and -B MGB probes. cRNA standards were constructed using the MEGAshortscript T7 kit (Ambion, Austin, TX, USA) and spectrophotometrically quantified.

The viral load of RV positive samples were quantified by qRT-PCR as described in the manuscript published by Lu and coworkers [10]. The Eurogentec One-Step Reverse Transcriptase qPCR kit was used for preparation of the master mix as described above. The primerset and probe, located in 5'UTR, were added to a final concentration of 1 μM and 0.1 μM, respectively. cRNA standards were constructed based on the PCR product of sample 1 using the MegaScript kit (Ambion, Austin, TX, USA). Quantification was performed with a spectrophotometer at 260 nm and converted to the molecule number [11]. Tenfold serial dilutions, allowing detection in a range of 8.6 × 10^6 ^to 8.6 × 10^2 ^RNA copies were used. The RT-PCR assays were carried out on a ABI PRISM 7500 Sequence Detection System (Applied Biosystems, Foster City, CA, USA). An initial reverse transcription step was performed at 48°C for 30 min, followed by a denaturation step at 95°C for 10 min. Finally, an amplification step of 45 cycli at 95°C for 15 sec and 1 min at 60°C was completed.

## Results

A total of 102 EBCs were collected from volunteers showing a symptomatic respiratory infection. Seventy were collected during the winter and spring of 2008/2009 and 32 during the fall and first part of the winter of 2009/2010. The first group of participants consisted of 42 (60%) women and 28 (40%) men and a median age of 32 years (range 19 - 83 years) was observed. The second group existed of 18 (56.3%) women and 12 (37.5%) men, with a median age of 29 (range 9 - 46 years). Age and gender was missing for 2 participants of the second group. In total, 52% of the participants were between 20-30 years old. Only 6% were younger than 20 years old and 3% were older than 70 years. In totality, 80 patients (78.4%) were already feeling ill for 1 to 7 days at the day the sample was obtained. Seven volunteers (6.8%) were symptomatic for 8 to 14 days and 9 participants (8.8%) were already ill for more than 14 days at the day of sample collection. Data on the duration of symptoms was lacking for 6 patients. Almost all volunteers experienced at least 2 symptoms except for two patients (Table 1). Forty-seven (46.1%) volunteers complained about a constant runny or stuffy nose, 43 (42.2%) had frequent sneezing events and 38 (37.3%) participants had a serious sore throat (Table 1).

### Viral screening

In a first part of the study, we collected 70 EBCs. Screening of the EBCs for 14 respiratory viruses (Table 2), showed 5 RV (7.1%) positive samples (Table 3). In a second part, we collected 32 EBCs from patients that presented themselves to their general practitioner. Two of these EBCs were positive for one of the 14 investigated respiratory viruses, 1 for RV and 1 for InfB. To inspect the detection rate of respiratory viruses in the condensate, a NS was taken from this second group of volunteers for comparison. In 15 out of 32 NS (46.8%), one or more viral pathogens were isolated. Viral screening of the NS resulted in the detection of RV, InfA (subtype H1N1) and HRSV-B. Quantification of the HRSV-B viral load demonstrated for samples 72 and 101 viral titers of 8.0 × 10^4 ^RNA copies/ml and 6.8 × 10^7 ^RNA copies/ml respectively. The RV RT-PCR assay did not allow the quantification of all samples that tested positive for RV by PCR (Table 3). Presence of the same pathogen in both the EBC and the NS was confirmed for only 1 sample: sample 71, which tested positive for RV in both the EBC and the NS. For sample 81, RV was detected in the NS and analysis of the EBC demonstrated an InfB infection.

**Table 3:** Viral screening of EBCs and NS

Patient	EBC	NS	Viral load in NS RNA copies per ml (Mean Ct ± SD)	Days of illness at time of collection
1	RV	-	ND	1
2	RV	-	ND	1
3	RV	-	ND	1
6	RV	-	ND	2
47	RV	-	ND	2
71	RV	RV	8.8 × 10^4^ (33.22 ± 0.34)	2
72	N	HRSV-B & RV	8.0 × 10^4^ (40.74*) 1.8 × 10^5^ (31.96 ± 0.06)	5
73	N	RV	3.8 × 10^5^ (30.86 ± 0.73)	4
74	N	RV	1.5 × 10^6^ (28.67 ± 0.29)	2
75	N	RV	ND	4
76	N	RV	ND	6
79	N	InfA (H1N1)		1
80	N	RV	7.9 × 10^5^ (29.66 ± 0.23)	1
81	InfB	RV	ND	14
83	N	InfA(H1N1)		2
87	N	RV	ND	1
89	N	RV	2.0 × 10^7^ (25.81 ± 0.11)	2
95	N	RV	4.0 × 10^6^ (28.53 ± 0.12)	2
96	N	RV	6.0 × 10^6^ (27.74 ± 0.82)	2
101	N	HRSV-B	6.8 × 10^7^ (29.18 ± 0.12)	3

RV = Rhinovirus, RSV-B = Respiratory Syncytial Virus B, InfA = Influenza A, InfB = Influenza B, N = Negative, - = No sample available, ND = viral load could not be determined.

SD = standard deviation * single detection

### Viral generation rate

For EBC samples that were collected in the fall and winter of 2009/2010, measurements with the ECoVent in combination with the RTube™ were performed for 13 of the 32 collections. A mean volume of 763.5 litre air (range 103.8 - 999.9) was exhaled during 5 min and volunteers breathed with a mean breathing frequency of 20.8 (range 18.2 - 30.7) times per minute. Only 1 EBC (Table 3, sample 81) was positive for InfB when using the RTube™ in combination with the EcoVent. In theory, the viral generation rate (number of viral RNA copies exhaled per minute) can be predicted by quantification of the exhaled viral load. Then, an estimation of the RNA copies per litre exhaled air or per minute can be calculated. Quantification of the exhaled InfB would allow us to predict the generation rate for this virus. Due to insufficient sample volume, we could not determine the number of RNA copies in the sample.

## Discussion

Collection of exhaled breath condensates is a novel and non-invasive method for obtaining samples of the upper respiratory tract. The collection of EBC is easy to perform and can be conducted in a home environment. This method is much more agreeable for the patient when compared to the unpleasant and invasive collection of nasal swabs, BAL, aspirates, etc. This aspect renders the method very attractive for routine laboratory diagnostics of viral infections. Most studies that perform breath analyses for viral detection use modified face masks, with a removable central region in electret or a removable Teflon filter on which exhaled particles impact [12-14]. With the RTube™ collection device, aerosolized particles of the airway lining fluid are precipitated into a condensate when the breath is cooled which serves as an immediate starting point for molecular testing.

Until now, this is the study with the largest subset of volunteers that investigated EBC as a specimen for the detection of respiratory viruses. Previous studies reported the inclusion of a limited subset of participants and investigated the presence of a limited number of viruses in the breath samples. The study performed by Fabian and colleagues, included 12 volunteers [12]. Huynh and co-workers recruited 9 volunteers for exhaled breath sampling [13]. In the study by Stelzer-Braid et al., 50 EBCs were analysed [14] and St-George et al. report the participation of 12 adults [15]. These studies have focused on the detection of InfA and -B, PIV1-3, HRSV and HMPV, while we have screened the samples for a panel of 14 commonly circulating respiratory viruses. Based on the analysis of 99 EBCs (3 EBCs were excluded), our results support the exhalation of RV and InfB in 7% of our samples. Since many of the volunteers had already been experiencing symptoms for 1 to 7 days, we initially presumed that they were already recovering from the infection and were no longer exhaling the virus. For common cold infections it is suggested that a person may already be infectious for 1 or 2 days before experiencing any symptoms. However, in a second part of our study we started collecting EBCs in parallel with nasal swabs from patients presenting themselves to their medical doctor, 1 to 3 days after onset of symptoms. Only for 1 condensate the same pathogen was detected in both the EBC and the NS. The detection rate for respiratory viral pathogens in the NS was 46.8% which is much higher than the 7% detection rate in the EBCs. The low detection of virus positive condensates can therefore not be attributed to the fact that volunteers were no longer infectious. The discrepant detection rate between samples may also be explained by different severity of respiratory infection, since comparator samples were of different parts of the respiratory tract. Patients that delivered a positive NS may have possibly suffered from an upper airway infection whereas EBC positive volunteers may have experienced a more advanced, lower respiratory tract infection. However, the effect of nasal inhalation on EBC collection, guiding formed particles in the upper respiratory tract to the lower compartments, in stead of oral inhalation was not investigated. Patients with positive EBC samples were experiencing symptoms for maximum two days at the time of collection. However, this was not different for 7 patients with positive NS. Six patients that provided positive NS were experiencing symptoms for a longer period at the time of collection (Table 3). In the group of volunteers that provided an EBC negative or EBC and NS negative sample, the manifestation of symptoms were reported ranging from 1 day to more than two weeks. When reported symptoms were compared between EBC positive patients (7) and NS positive patients (15), 27% and 33% in the positive NS group experienced shivering and muscle pain whereas this symptom was not indicated by any patient of the EBC positive group. In all groups fever, headache, watering eyes, stuffed nose, frequent sneezing, sore throat and coughing were reported.

Volunteers were not diagnosed with other pathogens before participation in the study. Since we did not test these samples for other than viral pathogens, we can not exclude the possibility that some of the negative NS are positive for bacteria or other pathogens causing respiratory illness. Recently, one study reported a detection rate of 5% for influenza in EBC [15]. This is in the same range of the detection rate that we report for respiratory viruses in general. Other studies with a limited number of patients, describe a markedly higher sensitivity of 33 to 36% [12-14] but the higher percentage may be due to the low number of participants subjects were included [12]. Remarkably, the studies reporting this higher detection rate used collections masks, while the study using the RTube™ reported comparable findings. Face masks consist of electret which trap viruses based on permanently charged fibres [13]. In addition, the Teflon filter has 2 μm pores which will retain all larger particles. Possibly, the lower detection rate can partly be explained by the fact that the RTube™ is manufactured in polypropylene and does not possess a virus attracting and filtering feature like the aforementioned materials.

The qRT-PCR developed by Lu and coworkers for the detection of RV, did not allow the assessment of the viral load present in the EBC samples [10]. Also for 4 NS, the viral titer remained undetermined, probably due to the limited sensitivity of the assay. For diagnosis, more sensitive methods might be necessary to detect respiratory viruses present in EBC since it is unpredictable how diluted the viral particles in the specimen are. Recently, nested qRT-PCR assays have been developed to allow a more sensitive detection of viruses in aerosols [16].

Also person-dependent factors, such as the number of particles produced, the exhaled volume and the age of the patient, have been suggested to play an important role for exhalation of viral particles. The participants that were recruited in the study of Fabian and coworkers were 12 years of age and older [12]. For hospitalized children a much higher rate of virus positive samples is reported [14]. In our study, the majority of volunteers were between 20 and 30 years old. Only two children less than 10 years and 3 elderly people (> 70 years) were included. One of the children tested positive for InfA in the NS, but the infection was not confirmed in the EBC.

For influenza, an exhaled generation rate of <3.2 to 20 influenza RNA copies per minute was predicted by quantifying the virus aerosols that impacted on a removable Teflon filter of a collection mask [12]. We used the RTube™ in combination with the ECoVent, that allowed the registration of additional ventilation parameters such as breathing frequency and exhaled volume. In this way, when the number of RNA copies in the EBC is quantified, the amount of viral particles that are exhaled per litre or per minute can be estimated. Unfortunately, we were not able to predict a virus generation rate for InfB since viral load remained undetermined.

Although an inventive, new and promising method, EBC collected by the RTube™ does not appear to be appropriate for diagnosis of respiratory infections. Nonetheless, this method may provide an alternative for current sample procurement for epidemiological studies of circulating viruses. This technique also confirms the observation that viruses are able to disseminate through normal breathing, particularly RV.

In addition, EBC collection from patients during respiratory infections may be further investigated for biomarker patterns. In calves that were experimentally infected with bovine RSV, an increase in leukotriene B_4_, indicating oxidative stress, was observed. This increased level was also associated with the development of bronchial hyperresponsiveness [17]. In humans, a transiently elevated H_2_O_2 _level was observed during common cold infection. This marker returned to baseline values when volunteers recovered from infection. H_2_O_2 _has also been recognized as an interesting marker in asthma, where it is associated with chronic lower airway inflammation [18]. In InfA infected volunteers, an increased CO level was observed during upper respiratory infection. This observation might imply that CO is an indicator of airway inflammation or represents one of the host defence mechanisms against viral infection [19]. Therefore, a better identification of the biomarker signature in condensates of individuals experiencing a viral infection might imply interesting findings towards the identification of markers reflecting inflammation or antiviral protection. This may contribute to the biomarker profiles established for diseases like asthma and COPD, for which viral infections are suggested to trigger or exacerbate symptoms [20].

## List of Abbreviations

AdV: Adenovirus; BAL: Brochoalveolaire lavage; COPD: Chronic Obstructive Pulmonary Disease; CoV: Coronvirus; EBC: Exhaled Breath Condensate; HMPV: Human Metapneumovirus; HRSV: Human Respiratory Syncytial Virus; InfA: Influenza A; InfB: Influenza B; NS: Nasal Swab; PIV: Parainfluenzavirus; RV: Rhinovirus; qRT-PCR: quantitative Reverse Transcriptase Polymerase Chain Reaction.

## Competing interests

The authors declare that they have no competing interests.

## Authors' contributions

LH designed, executed and coordinated the study. SDC contributed in the sample acquirement and laboratory analysis. EK, PM and JV participated in the execution of the study and added helpful suggestions during preparation of the manuscript. PN cooperated in this study and participated in the recruitment of volunteers and sample collections in a clinical setting. RDR, IT, MS work as medical doctors at the Medical Center of the University Leuven and supported our study by recruiting volunteers for our study. All author have read and approved the manuscript.
